# The role of computer technology in motion tracking method of basketball shot

**DOI:** 10.1371/journal.pone.0343758

**Published:** 2026-03-19

**Authors:** Weimin Cheng, Zhuoli Yu, Weidong Cheng

**Affiliations:** 1 Department of Physical Education, Dongshin University, Naju, South Korea; 2 Criminal Justice School, Zhongnan University of Economics and Law, Wuhan, China; 3 Department of Physical Education, Cyberspace Security University of China(CSUC), Wuhan, China; İzmir Democracy University: Izmir Demokrasi Universitesi, TÜRKIYE

## Abstract

With the rapid development of modern science and technology, with the gradual maturity of computer-aided training system, it has also been applied in sports training, and has achieved good results. The application of new technology can not only clearly see the mastery of sports technical skills and the training level of athletes, but also provide a more systematic basis for the next training plan of trainer system, and provide a set of scientific and reliable guidance for the development of athletes ’ sports potential. However, in complex scenes, there are some problems, such as occlusion object interference, which affect the tracking accuracy of target action. To address this problem, a basketball shooting action tracking method for complex scenes is proposed. The method first integrates the imaging system into a trackable invariant feature-fusion framework, allowing motion information in complex environments to be extracted more reliably. Through the visual measurement system, the motion of the target in the scene is detected, and the spatial node corresponding to the detected action is treated as the initial tracking point. An ant colony algorithm is then employed to construct an ant-colony tracking model, enabling robust tracking of the basketball shot-release motion under complex scene conditions. Simulation results demonstrate that the proposed method achieves strong tracking performance, with high accuracy, reduced tracking deviation, and improved preservation of motion details.

## 1. Introduction

With the rapid development of science and technology, motion tracking has become an important research topic in the field of computer vision and has attracted extensive attention in recent years. Motion tracking integrates navigation technology and human–computer interaction technology [1].

Real-time target locking and intelligent monitoring technologies can rapidly identify continuous or intermittent target motions within a complex background and determine their trajectories, enabling visual analysis of movement patterns and parameter evolution. In the context of basketball, however, the complexity of the background often causes the extracted 3D motion information to be disturbed by environmental occlusion or incorrect pixel data, leading to a reduction in tracking accuracy. To achieve reliable real-time tracking of basketball shooting actions and to improve motion tracking performance, researchers both domestically and internationally have explored various tracking methods designed specifically for basketball shooting movements in complex scenes. Liu Hui [2] employed a direct linear transformation (DLT) method to label the three-dimensional coordinates of motion nodes, and used a machine learning system to estimate the confidence intervals of these nodes. Through an AI-based CI identification system, the motion analysis curve of the target was obtained, enabling basketball shot-release tracking under complex scene conditions. However, this method still suffers from poor tracking performance.

Du Gang [3] enhanced the initial anchor points of target motion using a Wireless Sensor Network(WSN) node localization algorithm. By improving the signal coverage in low-frequency or invalid regions of complex scenes and ensuring high-accuracy alignment of longitudinal and transverse coordinates, the method effectively optimizes both the coordinate dimensions and network signal quality. A Lagrangian interpolation function was then used to predict the subsequent trajectory of the anchor points, thereby enabling basketball shooting action tracking in complex environments.

Zhang Weijun [4] and colleagues distinguished target-related information from background priors and established a pixel-level motion model. This model not only extracts movement patterns through correlation filtering but also estimates target deformation based on highly discriminative observations. By feeding scene-specific motion features into the model, the method achieves basketball shooting motion tracking in complex scenes. Nevertheless, these two approaches still exhibit limitations in tracking accuracy.

In order to solve the problems in the above methods, a basketball shot shot motion tracking method in complex scenes is proposed.

## 2. Construction of trackable invariant feature fusion space

As a hand movement with high tracking value in sports training, the basketball shooting action consists mainly of three components: elbow extension, wrist flexion, and fingertip release. Under standard conditions, an optimal shooting motion requires approximately a 90° elbow angle of the shooting arm, a 45° elbow angle of the supporting arm, and less than 90° of wrist dorsiflexion. A proper shooting technique not only improves shooting accuracy [5] but also reduces the likelihood of hand and wrist injuries. Therefore, the shooting motion is considered a key target for motion tracking and for providing posture guidance to athletes throughout the training process.

Current motion tracking methods still suffer from occlusions and pixel-level recognition errors, especially in complex background environments. To mitigate the tracking errors induced by such challenging scenes, an integrated imaging system has been proposed [6]. Building upon this system, corner features in the tracking background are extracted [7], while line features [8] together with scale features are incorporated as pixel-level descriptors for image registration. These combined features construct a trackable and invariant feature-fusion space that enables a substantial reduction in tracking errors.

The integrated imaging system is combined with an array optoelectronic reconnaissance system [9]. The speckle sensing system with attitude perturbation compensation platform is often applied to pixel space transformation of complex scenes because of its superior functions of complex scene reconnaissance and optical axis perturbation. It can scale the environment and improve the positioning accuracy and tracking accuracy of arbitrary moving or non-moving objects in the scene. Considering the diversity of complex scenes, the integrated imaging system starts from the corner and line aspects to extract the corner and line scale features of the athlete’s environment. The expression of the array photoelectric reconnaissance system is as follows:


$$R=\left[\ln\left(\frac{t(z_{m})}{y(z_{n})}\right)\right]h_{o}$$ (1)

Where, *R* represents the confusable similarity measure information; $t(z_{m})$ Denotes the rotational scale transformation parameter; *y* Represents the probability of spurious track elimination; $z_{n}$ Represents the starting time of model reconnaissance; $h_{o}$ Denotes the stop time of model reconnaissance. The formula does not originate from a physical model but is defined in this framework as a similarity measure designed to combine the rotational–scale transformation parameter and the spurious–track elimination probability.

In this study, the degree of optical–axis drift is quantified using the following task-specific perturbation measure:


$$P=\frac{c^{\prime }}{{\left\|yf_{1}-Of_{2}\right\|}^{2}+\partial }$$ (2)

where *P* denotes the drift measure of the optical axis, $c^{\prime }$ represents the initial optical–axis magnitude, *y* and *O* denote the initial position and the reference orientation of the optical axis, respectively, $f_{1}$ and $f_{2}$ represent the corresponding frame–wise pose correction references, and ∂ denotes the perturbation statistic used for compensation. The above expression is not derived from a physical model but is defined as a robust metric for estimating optical–axis perturbation under the proposed framework.

In this study, the corner–scale feature is defined as


$$L=\exp\left\{\sum_{i\neq 0}\beta_{i}\frac{q}{g(I_{n}-I_{m})}\right\},$$ (3)

where *L* denotes the fused corner–scale response, $\beta_{i}$ represents the robustness weight of the *i*-th corner feature, *q* denotes the Harris-based corner strength, $g(I_{n}-I_{m})$ describes the inter–frame gradient constraint between the corner points of the two consecutive frames, and $I_{n}$ and $I_{m}$ represent the corresponding image intensities. This expression does not originate from a closed-form derivation; rather, it is a task-specific definition inspired by classical log–ratio feature representations and scale–space fusion principles commonly used in corner detection and tracking.

The view–scale feature corresponds to pixel frames arranged sequentially along the temporal axis of the image sequence, where the spatial position of each pixel frame is recorded over time. Unlike the random distribution of neighboring pixel frames in a static histogram, the scale features associated with the athlete’s environment form a meaningful optical flow field because basketball shooting exhibits continuity in both the time and frequency domains [10]. This temporal coherence strengthens the correspondence between adjacent pixel frames and improves the stability of image registration [11].

The complexity of the expression for the spatial position of the pixel frame is as follows:


$$Y=\frac{1}{\sqrt{2}\sin^{2}\theta (u)}\;exp\left\{-\frac{s^{2}}{2\omega (dt_{r})}\right\}$$ (4)

Where, *Y* represents the joint degree of scale features; *θ*, *u* denotes the individual difference degree of frame per pixel; $s^{2}$ represents the spatial location fit; *ω* represents optical flow volume; *d* denotes time domain continuity; $d_{r}$ denotes continuity in frequency domain.

The formula for determining the frame correspondence of neighborhood pixels is as follows:


$$J=\frac{1}{\varpi }\int_{-\infty }^{+\infty }y_{i}(w)-\tau^{2}$$ (5)

Where, *J* represents the desired correspondence; ϖ $y_{i}$ Denotes actual correspondence; *w* Represents the pixel frame neighborhood information; *τ* Represents the Euclidean distance of the neighbor pixel frame.

As the evolution space of complex scene, the trackable invariant feature fusion space fully reflects the background features of the environment where the target action is located. The corresponding spatial position of each pixel frame is mapped to the fusion space, and the image registration of the current scene is realized. At the same time, the invariant feature fusion space whose tracking error is not valid in the complex scene is established, which lays a solid foundation for tracking the action of basketball shooting. The corresponding spatial location mapping formula of pixel frames is as follows:


$$K=\frac{1}{2}\sum_{j\neq 0}^{4}\left[g_{n}(j)-g_{n-1}\right]$$ (6)

Where, *K* denotes the univariate mapping sequence; $g_{n}^{\;j}$ represents the multivariable interactive mapping sequence; and $g_{n-1}$ describes the mapping ratio corresponding to the spatial location of the pixel frame along the X-axis and Y-axis.

The expression for the fusion space is as follows:


$$G=\frac{(e+i{)}^{2}}{k_{n}}$$ (7)

Where, *G* represents the fusion threshold in the horizontal direction; $k_{n}$, *e* Denotes the fusion threshold in the vertical direction; *i* Represents the spatial weight.

The expression for the trackable invariant feature fusion space is as follows:


$$E=(\nu +|\gamma |+\delta )\times p^{2}$$ (8)

Where, *E* represents the mechanical inertia parameter; *ν* *γ* denotes the dimension of spatial distribution; *δ* represents the number of feature variation; $p^{2}$ denotes the coincidence degree of complex scenes.

## 3. Detect the target motion action that exists in the space

To effectively track basketball shooting actions and enhance the quality of technical training, it is essential to first identify the target motion existing in the scene based on a trackable invariant feature-fusion space and treat it as the primary object for visual tracking and pattern recognition [12]. The CMU and Sarnoff vision measurement systems [13], which employ multiple cameras arranged in both single-mode and multi-mode configurations over a wide angular range, provide an important reference. These systems not only adapt well to the invariant feature-fusion space used for motion traceability, but also induce a camera-fusion segmentation space. As a result, moving objects within the scene—such as the basketball, backboard, and court surface—are segmented from the background through conventional weighting mechanisms and assigned to a foreground distribution, enabling more accurate motion tracking in complex environments.

However, in the fused feature space, moving objects such as athletes and basketballs are elevated from conventional distribution weights to priority distribution weights under the effect of background enhancement, thereby forming the spatial foreground. This foreground–background segmentation increases the contrast between moving and non-moving elements in complex scenes, which significantly improves the detection rate and the generalized recognition of target actions. The formulation of the vision measurement system is expressed as follows:


$$A=\sum_{i,j\in [1,1]}^{2}a_{ij}\left(|l_{0}-l_{ij}|-\Delta \varsigma \right)$$ (9)

Where, *A* represents the pixel frame density; $a_{ij}$, $l_{0}$ Denotes the removal rate of isolated noise points; $l_{ij}$ Represents the edge details of target action; Δς Represents the color projection histogram.

The formula for background reduction for non-moving objects is as follows:


$$M=\sum \frac{1}{m_{1}(S)+m_{2}(s)}$$ (10)

Where, *M* represents the initial distribution weight of the non-moving object; $m_{1}$, *S* Denotes the unit vector of universal gravitation; $m_{2}$ Denotes the strength of attraction.

The formula for background reinforcement of a moving object is as follows:


$$V=\frac{1}{2e_{ij}}min\left\{\frac{\sum_{i,j\in [0,1]}c_{i}c_{j}-\nu^{2}}{g_{ij}^{2}}\right\}$$ (11)

Where, *V* represents the initial distribution weight of the moving object; $e_{ij}$, $c_{j}$ denotes the freedom of the moving object; $c_{j}$ represents the measurement bias of accelerometer; $\nu^{2}$ represents the attitude angle of the moving object; $g_{ij}^{2}$ denotes the middle value of Kalman gain.

The expression for the spatial foreground is as follows:


$$F=v_{nm}\left(\varepsilon_{1}\cap \varepsilon_{2}\right)$$ (12)

Where, *F* represents the introduction of dynamic weight criteria; $b_{nm}$, $\varepsilon_{1}$ denotes the upper bound of the spatial foreground value; $\varepsilon_{2}$ denotes the lower value bound of the spatial foreground.

The expression for the spatial backshot is as follows:


$$Q=-\Delta t_{e}\times \varphi$$ (13)

Where, *Q* denotes the static weight introduced into the model standard, and $-\Delta t_{e}$ and φ represent the interaction factors associated with non-moving objects.

The segmented trackable invariant feature-fusion space concentrates the basketball shooting action within the spatial foreground. The dynamic parameters corresponding to the shooting motion in this spatial foreground are then identified through the dynamic sensing module, enabling effective detection of the target action.

The dynamic parameters of basketball shooting action can be obtained from the dynamic equation derived from the athlete’s long-range shooting trajectory, which is as follows:


$$B=\sum_{n\neq 0}\left\|v_{0}-v_{n}\right\|+\sum_{m\neq 0}\left\|z_{0}-z_{m}\right\|$$ (14)

Where, *B* represents the trajectory of basketball behavior; $v_{0}$, $v_{n}$ represents the movement space of the athlete’s upper limbs; $z_{0}$ represents inertia loss of athletes; $z_{m}$ denotes the error interference factor.

The expression of the power sensing element is as follows:


$$Z=\frac{\sqrt{y_{x-1}-\frac{y_{x-2}}{2}+(y_{REF}-y_{u})}}{\psi }$$ (15)

Where, *Z* represents azimuth perception; $y_{x-1}$, $y_{x-2}$ represents angle perception; $y_{REF}$ Stand for pose perception; $y_{u}$ represents sensing data sequence; ψ Represents the distribution sequence of kinetic parameters.

## 4. Motion tracking

The spatial node at which the target action is detected is used as the initial tracking point, and an ant colony algorithm [14] is employed to construct an ant colony–based tracking model for basketball shooting motion. The ant colony algorithm, also known as ant colony optimization (ACO), was first proposed by Marco Dorigo in 1991 based on the positive-feedback mechanism in which real ants deposit pheromones while foraging to efficiently locate food sources [15]. According to this principle, an ant colony exhibits intelligent behaviors such as searching for resources and selecting optimal paths, and the planning capability derived from these behaviors has been applied across numerous fields. In this study, the motion tracking process is designed based on the core concept of the ant colony algorithm. The athletes are modeled as ants distributed across the court, the basketball hoop serves as the target of the search, and the basketball shooting motion corresponds to the colony’s optimization process.

Based on the motion detection results obtained in the spatial foreground, the initial position of the target action can be determined [16–19]. Considering the one-to-one correspondence between each ant in the colony and a specific motion target, the start and end positions of the target action can be regarded as two fixed coordinates for global tracking. By combining these coordinates with the WGOA tracker, an ant colony–based tracking model can be established. The ant colony algorithm can be formulated as follows:


$$N=\int_{0}^{+\infty }d_{i}(f_{a}-w_{\beta }f_{b})/(P_{\alpha }^{\prime }-P_{\alpha +1}^{\prime })$$ (16)

Where, *N* represents the number of ant colonies; $d_{i}$ and $f_{a}$ denote the association distribution within the colony; $w_{\beta }$ represents the inertia weight of the ant colony; $f_{b}$ denotes the pheromone concentration in the optimization process; $P_{\alpha }^{\prime }$ is the constraint parameter of the optimization; and $P_{\alpha +1}^{\prime }$ represents the update variation in the ant colony optimization process.

The expression of Whale–Grasshopper Optimization Algorithm (WGOA) tracker is as follows:


$$H=-10log\left(-0.5\sqrt{\frac{1}{\mu }\sum_{k\neq 0}x_{k}^{2}}-10sin(2\phi v_{r})+\kappa^{2}\right)$$ (17)

Where, *H* represents the three-point line on the basketball court; *μ* and $x_{k}^{2}$ denote the initial values of the shooting-hit probability; *ϕ* represents the tracking coefficient associated with multi-sensor information; $v_{r}$ denotes the trajectory of the basketball shooting motion; and $\kappa^{2}$ represents the behavioral deviation of the motion.

The ant colony tracking model can be expressed as follows:


$$C=\frac{1}{2000}\sum_{j\neq 0}\theta_{j}^{2}-\prod_{u\neq 0}tan\left(\frac{q_{i}-q_{j}}{\sqrt{g_{u}}}\right)+e_{\alpha }$$ (18)

Where, *C* represents wind advection; $\theta_{j}^{2}$ and $q_{i}$ denote the gravitational acceleration acting on the basketball; $q_{j}$ represents the horizontal convergence speed of the basketball shooting motion; $g_{u}$ denotes the frame-drawing test period; and $e_{\alpha }$ represents the default tracking duration of the model.

## 5. Methods and analysis

In order to verify the overall effectiveness of the proposed basketball shooting motion tracking method in complex scenes, it is necessary to conduct simulation tests.

**Fig 1:**
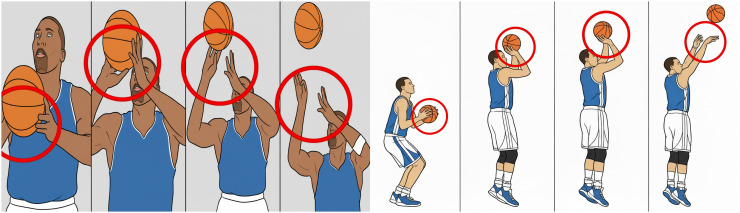
Tracking effect of the proposed method.

**Fig 2:**
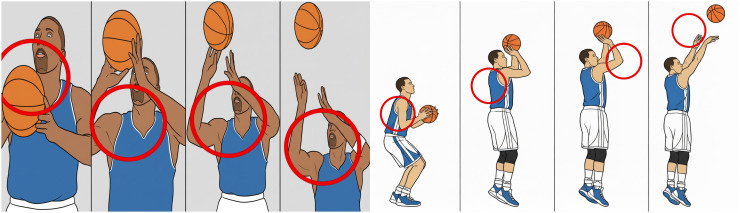
The tracking effect of the method in reference [2].

**Fig 3:**
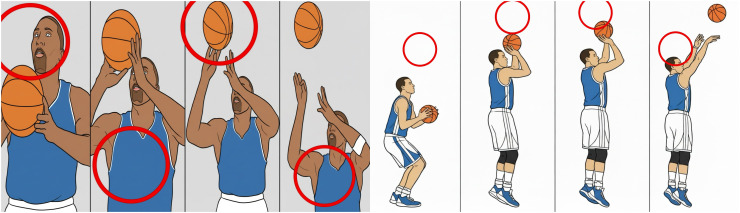
The tracking effect of the method in reference [3].

Two basketball players were randomly selected to perform shooting actions, and an image of each action variation was captured every 5 seconds. Four successfully extracted frames representing the dynamic changes in the shooting motion were used as test samples to evaluate the tracking performance of the proposed algorithm. These test samples are shown in Figure [1] Fig 1 Tracking effect of the proposed method [2], Fig 2 The tracking effect of the method in reference [3], Fig 3 Tracking effect of the method in referencecite Du 2020 [3].

All participants were fully informed about the purpose and procedures of the study prior to data collection. Written informed consent was obtained from all participants, and all data were collected and analyzed in accordance with ethical research standards.

As shown in Figure Figure [1–3], the proposed method achieves effective tracking of basketball shooting hand movements, maintaining accurate results at every time interval without deviation or target-loss issues. This improvement is primarily attributed to the use of a trackable invariant feature-fusion space prior to the tracking stage, which strengthens the representation of target motion in complex scenes. Consequently, the resulting tracking performance is more stable and reliable. In contrast, the methods described in [2] and [3] exhibit poor tracking performance for the shooting action across all time intervals, with frequent deviations and a high rate of target loss. The comparison clearly shows that the proposed approach significantly outperforms traditional methods in tracking basketball shooting motions [20].

**Fig 4:**
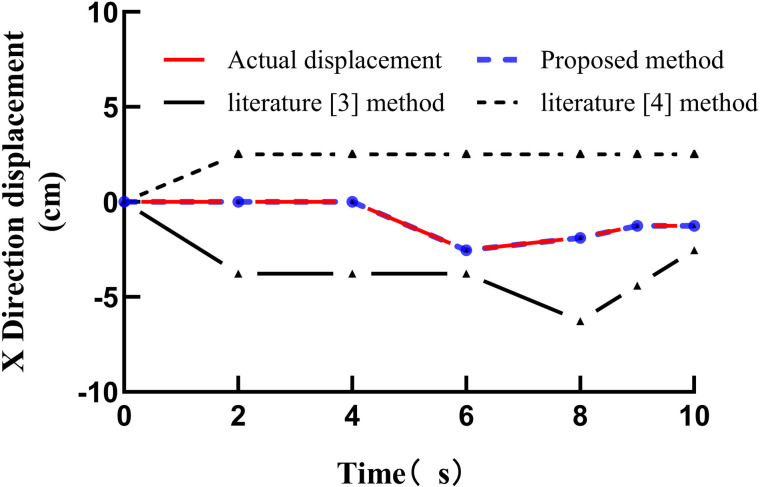
Actual displacements and tracking displacements of different methods in the X direction.

**Fig 5:**
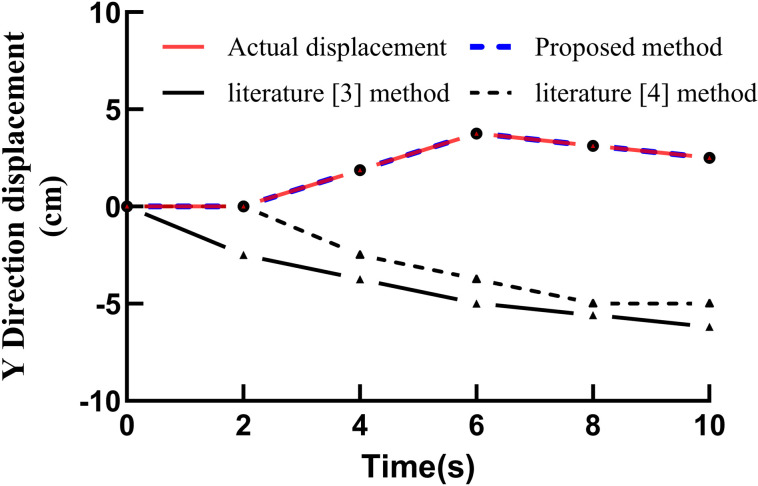
Actual and tracking displacements in the Y direction for different methods.

**Fig 6:**
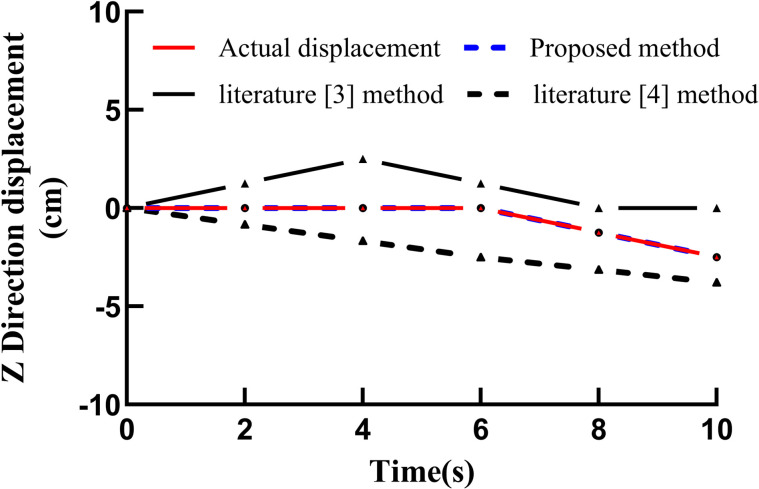
Actual and tracking displacements of different methods in the Z direction.

The proposed method, together with the methods described in [3] and [4], was applied to track the test samples, and the actual displacements and tracking displacements of the basketball shooting action in the X, Y, and Z directions were compared. The tracking and actual displacement curves obtained by the different methods are shown in Figure [4] Fig 4 Actual displacements and tracking displacements of different methods in the X direction, [5] Fig 5 Actual displacements and tracking displacements of different methods in the Y direction, [6] Fig 6 Actual displacements and tracking displacements of different methods in the Z direction.

As shown in figure bellow, the proposed method exhibits a high degree of coincidence between the actual and tracked displacements in the X, Y, and Z directions, indicating strong tracking performance. In contrast, the coincidence between the actual and tracked displacements obtained using the methods in [3] and [4] is noticeably lower, demonstrating that these methods yield inferior tracking performance. The comparison further confirms the practicability and effectiveness of the proposed approach [21].

## 6. Conclusion

Basketball has become one of the most popular sports in modern society, where competitive activities continue to flourish. To further improve players’ technical performance, this paper proposes a motion-tracking method for basketball shooting in complex scenes. Experimental results demonstrate that the proposed method achieves higher tracking accuracy and more stable tracking effects compared with traditional approaches. Based on these findings, incorrect shooting movements can be effectively identified and corrected, thereby guiding athletes toward more standardized shooting postures. In future research, a key challenge lies in achieving real-time monitoring of the shooting motion while simultaneously ensuring the robustness and efficiency of the tracking algorithm.
